# Vaccination against COVID-19 infection: the need of evidence for diabetic and obese pregnant women

**DOI:** 10.1007/s00592-021-01764-0

**Published:** 2021-06-28

**Authors:** A. Lapolla, M. G. Dalfrà, S. Burlina

**Affiliations:** Padova University, Padova, Italy

**Keywords:** Pregnancy, Diabetes, Obesity, Vaccine, COVID-19 infection

## Abstract

**Aim:**

The recent availability of vaccines against COVID-19 has sparked national and international debate on the feasibility of administering them to pregnant and lactating women, given that these vaccines have not been tested to assess their safety and efficacy in such women. As concerns the risks of COVID-induced disease, published data show that pregnant women who develop COVID-19 have fewer symptoms than patients who are not pregnant, but they are more likely to need hospitalization in intensive care, and neonatal morbidity. Aim of the present perspective paper is to analyze the current literature regarding the use of the vaccine against COVID-19 infection, in terms of safety and protection, in high risk pregnant women as those affected by diabetes and obesity.

**Methods:**

Analysis of literature about vaccination against COVID-19 infection in pregnancy.

**Results:**

The main health organizations and international scientific societies, emphasize that—although data regarding the use of COVID vaccines during pregnancy and lactation are still lacking—vaccination should not be contraindicated. It should be considered for pregnant women at high risk of exposure to COVID-19. For such women, the potential benefits and risks should be assessed by the healthcare professionals caring for them. A recent prospective study to test the immunogenicity and reactogenicity of vaccination with COVID-19 mRNA in pregnant and lactating women, has showed that SARS-CoV-2 mRNA vaccination triggers a robust humoral immunity in pregnant and lactating women; there was also evidence of an immune transfer to their newborn.

**Conclusions:**

We urgently need data on the effect of COVID-19 vaccination, in terms of maternal and fetal outcomes and vaccine related symptoms in high risk women during pregnancy and breastfeeding. It is important to run campaigns to promote vaccination, in particular in pregnant women at high risk to have severe COVID infection as those diabetics and/or obese.

The recent availability of vaccines against COVID-19 has sparked national and international debate on the feasibility of administering them to pregnant and lactating women, given that these vaccines have not been tested to assess their safety and efficacy in such women.

Aim of the present perspective paper is to analyze the current literature regarding the use of the vaccine against COVID-19 infection, in terms of safety and protection, in high risk pregnant women as those affected by diabetes and obesity.

The currently authorized Comirnaty (Pfizer) and Moderna vaccines are mRNA vaccines. They are rapidly degraded, they do not penetrate the cell nucleus, and they have revealed no harmful effects in pregnancy in experimental animals [1, 2]. At the end of 2020 Pfizer and Moderna reported, respectively, 23 and 13 pregnant women inadvertently enrolled in their clinical trials (12 and 6 women, respectively, in the vaccine group). The outcomes of pregnancy are under evaluation [1, 2]. Since vaccination during pregnancy is generally safe and effective, this would suggest that vaccines against COVID-19 may be administered too, though we do not currently have strong scientific and clinical evidence to support this action. It is therefore imperative to conduct a cost–benefit analysis, assessing the risks of COVID-induced disease to the mother and fetus, and the benefits deriving from vaccination.

As concerns the risks of COVID-induced disease, published data show that pregnant women who develop COVID-19 have fewer symptoms such as cough and muscle pain than patients who are not pregnant, but they are more likely to need hospitalization in intensive care, and neonatal morbidity is more frequent than among pregnant women without COVID [3–7].The data on maternal mortality vary, depending on the different populations studied: they generally indicate a rate below 2.5%, but cases are more frequent among African American and non-Hispanic Black women [3–10]. In this frame, there is need to emphasize that in the meta-analysis of Khalil et al. [6] 38.2% of 2567 pregnant women presenting symptoms of SARS-COV-19 were obese; furthermore, the data collected from eight United States Care Centers shows that prepregnancy obesity and gestational diabetes were prevalent in pregnant women hospitalized for SARS-COV-19 related illness, with respect of those hospitalized for delivery and found to have SARS-COV-2 ([7]. Moreover data reported by the Vaccine Safety Datalink relating to the surveillance of COVID-19 hospitalizations from 1 March to 30 May 2020 [11] showed that conditions such as prepregnancy obesity and gestational diabetes were more common among pregnant women hospitalized for COVID-19 than among pregnant women hospitalized for obstetric reasons (respectively, 44% vs 31% for obesity, and 26% vs 8% for GDM).

The main health organizations and international scientific societies, including the World Health Organization (WHO) [12], the US Food and Drug Administration (FDA) [13], the American College of Obstetricians and Gynecologists (ACOG) [14], the Royal College of Obstetricians and Gynaecologists (RGOG) in the UK [15], the European Medicines Agency (EMA) [16], and the Italian Obstetric Surveillance System (ItOSS) [17], all emphasize that—although data regarding the use of COVID vaccines during pregnancy and lactation are still lacking—vaccination should not be contraindicated. It should be considered for pregnant women at high risk of exposure to COVID-19. For such women, the potential benefits and risks should be assessed by the healthcare professionals caring for them. The decision should be made on a case-by-case basis, particularly taking into account the rate of the virus’s dissemination in the pregnant woman’s town or country of residence, and workplace [12–17]. As concerns women planning a pregnancy, only the ACOG recommends they be vaccinated (if they meet the criteria for prioritization for vaccination) and states that there is no need to delay pregnancy after completing the second dose of vaccination [14]. This is particularly relevant for diabetic women planning a pregnancy.

In this context Shimabukuro et al. [18] recorded data from the “V-safe after vaccination health checker” surveillance system, the v-safe pregnancy registry and the Vaccine Adverse Event Reporting System (VAERS), from 14 December 2020 to 28 February 2021, to verify the effect of mRNA COVID-19 vaccine in pregnant women. The “v-safe after vaccination health checker” identified 35,691 pregnant women, age 16–54 years, injection site pain was more frequent in pregnant with respect to non-pregnant women, other side effects as myalgia, fever, chills, headache were reported less frequently. The v-safe pregnancy registry enrolled 3958 pregnant women and 827 of them had a completed pregnancy of which 12.6% have a spontaneous abortion, 9.4% a preterm birth, 3.2% a baby small for gestational age, no neonatal deaths occurred. All the adverse pregnancy outcomes (including spontaneous abortion) were similar in frequency to those reported in studies involving pregnant women conducted before the SARS-COV-2 pandemic even if this comparison is limited by the unknown differences between this populations in terms of clinical and demographic characteristics. As stated by the authors the frequency of spontaneous abortion found do not reflects the real post-vaccination frequency because pregnant women might have been vaccinated after the period of the main risk in the first trimester and this is a limit of the paper. Then, there are no information about the clinical characteristics of the pregnant women (such as presence of diabetes or obesity) due to the data collection made by telephone-based survey and this represents a further limit of the study.

In addition to vaccination protecting women against Covid-19 during pregnancy, there are some evidence of transplacental transfer of SARS-CoV-2 antibodies. Flannery et al. [19] examined the association between maternal and neonatal SARS-CoV-2 IgG and IgM antibodies in plasma and cord blood in 1714 pregnant women at the time of delivery at the Pennsylvania Hospital (USA). SARS-CoV-2 IgG and IgM antibodies were detected in 83 of the 1714 women (6%); and there were IgG in cord blood samples of 72 (87%) of their 83 newborn. No IgM or antibodies were detected in the neonates born to seronegative mothers. Cord blood IgG levels correlated positively with maternal IgG levels (*r* = 0.86; *p* < 0.001). Interestingly, a placental transfer ratio higher than 1 was found not only in pregnant women with symptomatic SARS-CoV-2 infection, but also in those with asymptomatic infection; and this ratio increased with the time between the onset of infection and delivery. The authors concluded that their findings “demonstrate the potential for maternal-derived SARS-CoV-2 specific antibodies to provide neonatal protection from coronavirus disease.”

More recently, Gray et al. [20] conducted a prospective study to test the immunogenicity and reactogenicity of vaccination with COVID-19 mRNA in pregnant and lactating women, comparing them with non-pregnant controls and women who became infected with COVID-19 during their pregnancy. The study prospectively enrolled 84 pregnant, 31 lactating, and 16 non-pregnant women at two centers in the USA. Titers of the SARS-CoV-2 spike and receptor binding domain (RDB) IgG, IgM and IgA were measured: at the baseline; on administration of the second dose of vaccine; 2 weeks after the second dose; and at delivery, when umbilical cord sera were also tested for antibodies. As concerns mean gestational age at the time of receiving their first dose of vaccine, 13% of the women were in the first trimester, 46% in the second, and 40% in the third. The data collected were compared with those obtained in pregnant women 4–12 weeks after they had become infected with the virus. A questionnaire was also used to record post-vaccination symptoms. The pregnant and lactating women showed similar vaccine-induced antibody titers to those of the non-pregnant women (median: 5.59 pregnant, 5.74 lactating, 5.62 non-pregnant). All these titers were significantly higher than those measured in pregnant women who became infected with SARS-CoV-2 during pregnancy (*p* < 0.001). Interestingly, antibodies against the vaccine were found in umbilical cord blood and breastmilk samples. The second dose of vaccine prompted an increase in SARS-CoV-2-specific IgG in maternal blood and breastmilk, but not in SARS-CoV-2-specific IgA. The side effects were no different between the groups of women examined, and mainly included fever, injection site reactions, and fatigue. On the basis of these results, the authors concluded that SARS-CoV-2 mRNA vaccination triggers a robust humoral immunity in pregnant and lactating women that is significantly stronger than after natural infection with the virus. There was also evidence of an immune transfer to their newborn.

These two interesting recent studies warrant some comments.

The importance of the study by Flannery et al. lies in demonstrating that, while placental transfer ratios can vary, symptomatic or asymptomatic maternal infection prompts a good antibody production, and these antibodies are transferred to the newborn—so maternal vaccination could plausibly achieve the same results. Consistently with this assumption, the paper by Gray et al. demonstrates—for the first time—that the COVID-19 mRNA vaccines can induce a strong humoral immunity in pregnant women that is transferred to the neonate, thereby conferring both maternal and fetal benefits.

It is interesting that 8% of the women testing positive for COVID-19 infection in the population examined by Flannery et al. had diabetes, and 35% were obese. In the study by Gray et al., 4% of the pregnant women had gestational diabetes, 12% had diabetes, and 19% were obese, while 10% of the lactating women had experienced gestational diabetes, 10% were diabetic, and 23% were obese. Bearing in mind the risks associated with COVID-19 infection for pregnant women, the presence of obesity, diabetes and/or hypertension in such women, and/or their belonging to ethnic minorities known to be at greater risk of severe COVID infection, Intensive Care Unit (ICU) admission, and neonatal morbidity [3–11], these data support a compelling argument for vaccinating pregnant women with mRNA vaccines against SARS-CoV-2. It would be an important weapon for preventing SARS-CoV-2 in such women, especially if they have additional high-risk factors such as diabetes and obesity [3–17]. We have to remember that both diabetes (especially gestational diabetes) and obesity have become dramatically more common in recent years all over the world [21–23], and that these diseases can cause numerous maternal and fetal complications [21–24]. Unfortunately, as regards the risk conferred by diabetes and/or obesity during SARS-CoV-2 infection in pregnancy, few data are available at the moment, as previously reported [6, 7, 11].

Despite these interesting findings, we are aware that a number of issues need to be solved. Taking into account the reports from Gray et al. and Flannery et al., and bearing in mind that at least two weeks from complete vaccination are necessary for high efficacy of vaccines, and that transplacental transfer starts at about 17 weeks of gestation, the best time for vaccinating pregnant women could plausibly be the second trimester. Thus, possible episodes of fever can be avoided in the first trimester of pregnancy [1, 2, 25].

The published data demonstrate that higher levels of antibodies coincide with higher levels of protection of the newborn, but no data are available on how long this protection lasts.

High levels of type III interferon, IFN-*λ*, secreted by syncytiotrophoblasts is a main defense mechanism of placenta against virus infections [26]. In this context in primary human epithelial cells cultures the treatment with IFN-λ, has reduced the SARS-COVID-2 infection [27]. However studies are necessary to verify if the placenta is permissive to SARS-COV19 replication.Then, recent papers on the topic have shown, with high specific methods [28], that placentas from pregnant women infected with COVID-19 have inflammatory vascular and thrombotic features as vascular malperfusion with central and peripheral villous infartctions, fibrin deposition and chrorionic villitis and and intervillositis with inflammatory infiltrate of CD68 + , macrophages and T cells [29].The coronavirus is also capable of triggering pattern recognition receptors that prompt activation of the transcriptional program, thereby inducing cytokine activation in the form of IL-6, which has proved predictive of long-term complications in neonates, such as neuropsychiatric disorders [30].

Could maternal antibodies have a detrimental effect on the infant’s response to immunization? Even if this is hardly plausible, given the safety of vaccines currently used to protect children (against measles, chickenpox and whooping cough), follow-up studies need to be conducted on neonates of vaccinated women.

Are the antibodies transferred by lactation capable of protecting the infants against infection? The results obtained by Gray et al. indicate a preferential transfer of IgG from milk to the newborn, whereas the study by Pace et al. on natural SARS-CoV-2 infections show a preferential transfer of IgA [31]. Given the crucial role of IgG in breast milk in inducing neonatal immunity against HIV, and influenza [32], the exact role of IgG and/or IgA in neonatal protection remains unclear.

Further studies are needed to confirm these interesting data, examining large numbers of unselected pregnant women of different ethnicities and with different risk factors relating to SARS-CoV-2 infection (the study by Gray et al. considered women of primary healthcare workers from one city (Boston, USA)).

Then there is the matter of pregnant women’s acceptance of vaccination against COVID-19. Skjefte et al. [33] conducted an online survey on individuals of various nationalities, with 17,871 respondents from 16 countries. While 52% of pregnant women and 73.4% of non-pregnant women would agree to being vaccinated, and 62% of women (pregnant or not) intended to have their children vaccinated, there was a marked variability from one country to another. Predictors of vaccine acceptance were confidence in the safety and effectiveness of vaccines, and fear of COVID-19 infection. Carbone et al. at the University of Naples (Italy) conducted a questionnaire-based survey to assess the attitude to vaccination of pregnant (119) and breastfeeding mothers [34]. Most of the women (71.8%) disagreed with vaccination during pregnancy, and their main reasons for this was the condition of pregnancy itself.

So, it is imperative to run campaigns to promote vaccination, which should be tailored to the country concerned to obtain the widest possible consensus.

In the case of breastfeeding mothers, they can receive COVID-19 Vaccination without stopping lactation [17]. In fact the Academy of Breastfeeding Medicine state that: “during lactation it is unlikely that the vaccine lipid would enter the blood stream and reach breast tissue. If it does it is even less likely that either the intact nanoparticle or mRNA transfer into milk. In the unlikely event that mRNA is present in milk, it would to be expected to be digested by the child and would be unlikely to have any biological effect. While there is little plausible risk for the child, there is a biologically plausible benefit. Antibodies and T-cells stimulated by the vaccine may passively transfer into milk. Following vaccination against other viruses, IgA antibodies are detectable in milk within 5–7 days. Antibodies transferred into milk may therefore protect the infant from infection with SARS-CoV-2” [35].

Finally, as regards pregnant women with diabetes and/or obesity, the types of patient for whom vaccination should be strongly recommended it remains to be seen: those with type 1 and type 2 diabetes, all GDM, GDM requiring insulin therapy, or GDM associated with multiple risk factors? Should vaccination be strongly promoted for all women with pregestational diabetes planning a pregnancy, or would it be better to define a cluster of risk factors? For obese women, should vaccination be offered to patients planning a pregnancy, to all pregnant obese women, or only to the morbidly obese? We urgently need to resolve these issues, and therefore systematic and proactive data on the effect of COVID-19 vaccination, in terms of maternal and fetal outcomes and vaccine related symptoms in high risk women during pregnancy and breastfeeding are mandatory.

Whatever the approach taken, it is crucial for healthcare providers to give sound scientific advice to pregnant women, explaining the mode of action, the possible adverse effects, the safety and the efficacy of the vaccines against COVID-19 on the basis of what actually known.
